# Non-syndromic tooth agenesis in Latvian adolescent dental patients: a retrospective study with relevant literature review

**DOI:** 10.1007/s40368-024-00901-x

**Published:** 2024-06-06

**Authors:** D. Meistere, L. Kronina, A. Karkle, L. Neimane

**Affiliations:** 1 Department of Conservative Dentistry and Oral Health, Riga Stradins University, Riga, Latvia; 2 Riga Stradins University Institute of Stomatology, Riga, Latvia; 3 Baltic Biomaterials Centre of Excellence, Headquarters at Riga Technical University, Riga, Latvia

**Keywords:** Tooth agenesis, Congenitally missing teeth, Hypodontia, Oligodontia, Dental anomalies, Panoramic radiograph

## Abstract

**Aim:**

To investigate the prevalence of tooth agenesis and associated dental anomalies in Latvian adolescent dental patients and compare it to other European countries.

**Design:**

Cross-sectional study of 2692 11-to-14-year-old patients (39.9% males and 60.1% females) attending Riga Stradins University Institute of Stomatology with panoramic radiographs taken between August 2020 and September 2021. Patients with any genetic syndromes were excluded. Data on tooth agenesis (excluding third molars) and other dental anomalies were recorded.

**Results:**

The prevalence of tooth agenesis in Latvian adolescent dental patients was 9.3% with no statistically significant difference between genders (χ^2^ test, *p* = *0.472*). The most commonly missing teeth were mandibular second premolars, followed by upper lateral incisors and upper second premolars. There was a statistically significant association with the presence of other dental anomalies in tooth agenesis patients (*p* < *0.001*).

**Conclusions:**

This study found that the prevalence of non-syndromic tooth agenesis in Latvian adolescent dental patients was 9.3% with no statistically significant differences between the genders. Patients with tooth agenesis have a statistically significant possibility of the presence of other dental anomalies (*p* < *0.001*).

## Introduction

Congenitally missing teeth is quite a frequent condition. It is considered one of the most common congenital anomalies in humans (Souza-Silva et al. 2018). Congenitally missing teeth or selective tooth agenesis is the developmental absence of at least one tooth. Generally, hypodontia is the absence of up to 5 teeth, and oligodontia is the absence of 6 and more teeth. Anodontia is the most severe form of tooth agenesis with the absence of all teeth (Khalaf et al. 2014). Tooth agenesis can occur as part of a genetic syndrome or as a non-syndromic isolated trait (Al-Ani et al. 2017). It is reported that the overall prevalence of non-syndromic hypodontia is 6.4% (Khalaf et al. 2014). The aetiology of tooth agenesis is explained by numerous theories, and it is understood as an interplay between environmental and genetic factors (Al-Ani et al. 2017).

Patients with this condition have to undergo continuous orthodontic treatment and possibly high-cost future interventions (Dos Santos et al. 2022). Diagnosing tooth agenesis promptly is highly important (Hvaring and Birkeland. 2019). Several other dental anomalies are associated with tooth agenesis, for example, microdontia, changes in crown shape, and impacted maxillary canines (Khalaf et al. 2014). Patients' oral health-related quality of life is impacted in more severe cases of tooth agenesis. These children experience considerable psychological, social, and functional effects (Locker et al. 2010). Patients with tooth agenesis can also face aesthetic concerns. In addition, tooth agenesis should be considered a marker for the risk of neoplasms in adulthood (Ritwik and Patterson 2018). A statistical association between hypodontia and epithelial ovarian cancer has been found (Chalothorn et al. 2008). All these factors should show that tooth agenesis is a significant pathology and should not be overlooked.

There are no studies about tooth agenesis in the Latvian population. This study was designed to investigate the prevalence of this condition in adolescent dental patients and compare it to other European countries. In addition, associated dental anomalies were also investigated.

## Materials and methods

### Study sample

This cross-sectional retrospective study analysed all panoramic radiographs of 2717 11-to-14-year-old patients who attended Riga Stradins University Institute of Stomatology between August 2020 and September 2021. Twenty-five patients whose medical history revealed some genetic syndromes were excluded from this study. The sample size consisted of 2692 patients (39.9% males and 60.1% females). The mean age was 156 months (SD = 13 months).

The age of 11–14 years was chosen based on recommendations not to include patients younger than 9 or 10 due to possible delayed development of second premolars (later than the typical age range) (Rakhshan. 2015).

### Radiological analysis

Tooth agenesis and other dental anomalies were registered from the panoramic radiographs by the author with consulting co-author. Third molar hypodontia was not included in this study. If one patient had more than one panoramic radiograph, then a pre-treatment radiograph was used to confirm the diagnosis of tooth agenesis. The tooth was registered as congenitally missing if there was no sign of tooth crown mineralisation on the panoramic radiograph. If needed, dental anamnestic data were viewed to confirm that the tooth was not missing due to orthodontic extraction, caries, trauma, or periodontal disease. The tooth was not registered as missing if the panoramic radiograph and dental anamnesis did not provide sufficient information.

### Statistical analysis

For data analysis, statistical software program Jamovi 2.3 (*The jamovi project, Australia*) was used. Findings in this study were reported using descriptive statistical analysis. To find the association between tooth agenesis and gender as well as tooth agenesis and associated dental anomalies, Chi-square and Fisher’s exact statistical tests were applied.

### Ethical committee

This study was approved by Riga Stradins University’s Research Ethics Committee (Nr. 22-2/424/2021).

## Results

In this study, 2692 panoramic radiographs were analysed. The prevalence of tooth agenesis was 9.3% (*N* = 250).

The majority of patients (*N* = 2116; 78.6%) were referred for dental panoramic radiographs by orthodontists. One hundred and seventy (6.3%) patients were referred by paediatric dentists, and 97 (3.6%)—by maxillofacial surgeons. Three hundred and eight (11.5%) referrals were either from a different clinic or were not specified.

### Tooth agenesis

473 congenitally missing teeth were identified, most commonly in the mandible (*N* = 274; 58%). Out of all hypodontia patients, 120 (48%) had 1 missing tooth, 86 (34.4%)—2 missing teeth, 23 (9.2%)—3 missing teeth, and 9 patients (3.6%)—4 missing teeth. Oligodontia was noted in seven (2.8%) of all hypodontia patients. Mandibular second premolars were the most commonly missing tooth group, followed by maxillary lateral incisors and maxillary second premolars. All commonly missing teeth, including distribution in each quadrant (several tooth notation systems divide maxilla and mandibula into 4 quadrants (Erfan et al. 2022); this principle is used by Latvian dentists), are outlined in Table 1.

**Table 1:** Amount and distribution of congenitally missing teeth

Congenitally missing teeth	Total (N; %)	Maxilla		Mandible	
		*Quadrant I*	*Quadrant II*	*Quadrant III*	*Quadrant IV*
*Central incisors*	8 (1.7%)	0	0	5	3
*Lateral incisors*	**113 (23.9%)**	**56**	**42**	10	5
*Canines*	9 (1.9%)	4	5	0	0
*First premolars*	10 (2.1%)	3	4	2	1
*Second premolars*	**312 (65.9%)**	**39**	**38**	**119**	**116**
*First molars*	0 (0%)	0	0	0	0
*Second molars*	21 (4.4%)	3	0	8	10
*Total in each quadrant*		**105**	**89**	**144**	**135**

In bold—most commonly missing teeth

Although more prevalent in females than males (5.9% and 3.6% respectively), there was no statistically significant difference between genders and hypodontia (χ^2^ test, *p* = *0.472*).

### Presence of other dental anomalies

Out of all hypodontia patients (*N* = 250), 39 (15.6%) had at least 1 other dental anomaly. The association was statistically significant (Χ^2^(1, *N* = 2692) = 387, *p* < *0.001*). The majority of patients (*N* = 32) had only one other dental anomaly. The rest of the patients had two associated dental anomalies. Dental anomalies that were found were upper canine impaction, second premolar impaction, malformation (microdontic or peg-shaped) of the lateral incisor, malformation of wisdom tooth, canine transposition, supernumerary tooth, delayed development of second premolar and ankylosis of second primary molars which have permanent tooth successor. The most common dental anomaly was malformation of one upper lateral incisor (*N* = 15), followed by upper canine impaction (*N* = 9, a total of 12 canines) and malformation of both upper lateral incisors (*N* = 5).

## Discussion

This is the first and only study about tooth agenesis in Latvia. The present study shows the prevalence of non-syndromic tooth agenesis in 2692 11–14-year-old Latvian dental patients to be 9.3%.

In a 2014 meta-analysis done by Khalaf and co-authors, it is reported that the prevalence of tooth agenesis in Europe was 7% and that there were statistically significant differences by continent (Africa—13.4%, Asia—6.3%, Australia—6.3%, North America—5.0%, Latin America and the Caribbean—4.4%) (Khalaf et al. 2014).

For the present study, age range was set to 11–14 years. The inclusion of younger patients may result in false diagnoses because of possible delayed development of second premolars. The inclusion of older patients also may result in false diagnoses because of possible tooth extractions due to orthodontic treatment, caries, periodontal disease, or other reasons. The majority of European studies referred to Table 2 having quite a wide range of included patients’ age, making direct comparing tooth agenesis prevalence between countries difficult. Unified guidelines for diagnosing and reporting tooth agenesis are needed.

**Table 2:** The prevalence of tooth agenesis in some European countries since 1977

Country	Year	Sample size	Age	Population	Prevalence %
*Albania *(Vinjolli et al. 2023)	2023	779	8–30	Orthodontic patients	8.9
*Bosnia and Herzegovina *(Dzemidzic et al. 2020)	2020	4256	9–16	Orthodontic patients	3.42
*Croatia *(Drenski Balijaet al*. *2022)	2022	506	12–16	Orthodontic patients	7.5
*Croatia (Southern part) *(Badrovet al.2017)	2017	4430	6–15	Orthodontic patients	7.8
*Denmark *(Rølling & Poulsen. 2009)	2009	8138	9–12	Schoolchildren	7.41
*France *(Baron et al. 2018)	2018	551	18 or younger	Not specified	5.81
*Greece *(Pallikaraki et al. 2020)	2019	1200	7–17	Orthodontic patients	0.17; 6.42^b^
*Hungary *(Gábris et al. 2006)	2006	2219	6–18	Orthodontic and paediatric patients	14.69
*Iceland *(Magnússon. 1977)	1977	1116	8–16	Schoolchildren	7.9
***Italy***(Gracco et al. 2017)	2017	4006	9–16	Dental patients	9
*Italy *(Laganà et al. 2017***)***	2017	4706	8–12	Orthodontic patients	7.1
*Italy *(Marra et al. 2021)	2021	4000	7–15	Not specified	9.30
*Kosovo *(Reshitajet al*. *2019)	2019	3306	15–21	Dental patients	2.3
*Lithuania *(Trakinienė et al. 2013)	2013	824	10–39	Orthodontic patients	17.11
*North Macedonia *(Pop Acev & Gjorgova. 2014)	2014	8160	8–18	Orthodontic patients	7.52
*Norway *(Aasheim & Ogaard. 1993)	1993	1953	6, 9, 12, 15, 18, 21	Orthodontic patients	6.5
*Norway *(Haugland et al. 2013)	2013	500	12	Orthodontic patients	6.6
*Poland* (Kielan-Grabowska et al. 2019***)***	2019	674	6–15	Orthodontic patients	11.6
*Portugal *(Campoy et al. 2013)	2013	2888	7–21	Dental patients	6.1
*Romania *(Ţenţ et al. 2018)	2018	566	12–18	Not specified	3.18
*Slovenia *(Fekonja. 2015)	2015	2546	15–16, 25–26, 35–36, 45–46	Dental patients	6.9
*Spain *(Sola et al. 2018)	2018	2500	7–11	Dental patients	3.48
*Spain *(Tallón-Walton et al. 2010)	2010	1518	6–83	Dental patients	7.25
*Spain *(Varela et al. 2009)	2009	2108	7–16	Orthodontic patients	6.5

^a^ Oligodontia

The prevalence of tooth agenesis among European countries when the third molars are excluded varies from 2.3% to 14.69% (Table 2). Compared to other European populations, the results of this study show a higher prevalence of tooth agenesis. Higher prevalence could be explained by Riga Stradins University’s Institute of Stomatology being the country’s largest multidisciplinary dental and orthodontic clinic. Similar results are reported among the Italian—9.30% (Marra et al. 2021) and Albanian patients—8.9% (Vinjolli et al. 2023). The highest prevalence of tooth agenesis is reported in Hungarian orthodontic and paediatric patients—14.69% (Gábris et al. 2006). In Lithuania, a neighbouring country to Latvia, the prevalence is reported to be 17.11%, but this includes missing 3rd molars, which were excluded in the present study, and thus cannot be compared (Trakinienė et al. 2013). In 1997, Peltola with co-authors conducted radiological study of 14–17-year-old schoolchildren in Estonia where prevalence of missing teeth was 14%, but it was not specified if teeth were congenitally missing or extracted (Peltola et al. 1997). High prevalence was also noted in Polish orthodontic patients—11.6% (Kielan-Grabowska et al. 2019).

The sample size of the present study contained all 11–14-year-old patients who received a panoramic radiographical examination during the period of 13 months. These patients were referred to radiological examination from different departments. In other studies performed in European countries, the sample mainly consisted of orthodontic patients. This may be a reason why the prevalence of tooth agenesis in the present study is higher than average in Europe. In this case, it might indicate that some patients and their parents are not aware of normal dentition and its consequences on a child’s aesthetics, mastication, and well-being.

The aetiology of tooth agenesis is multifactorial involving genetic regulation and environmental factors. This can explain high variations of tooth agenesis prevalence between different countries or even continents. Environmental factors, such as exposure to toxins or substances, trauma, and infection during pregnancy, can influence tooth development (Al-Ani et al. 2017). Variations in environmental factors between countries or regions can contribute to differences in prevalence. Further research is needed to fully understand the complex interplay of genetic and environmental factors that contribute to the differences in tooth agenesis prevalence between countries. Differences in dental care access and awareness also may contribute to the detection and reporting of tooth agenesis.

Overall females are found to have a higher prevalence, but only a few studies show statistical significance (Khalaf et al. 2014; Rakhshan and Rakhshan. 2016). In the present study prevalence of tooth, agenesis was more common in females, but it was not statistically significant. This is in agreement with most of the studies conducted in European countries. Exceptions are reported in Danish and Norwegian studies where female predominance was statistically significant although no possible explanation was provided (Badrov et al. 2017; Haugland et al. 2013; Rølling & Poulsen. 2009). In the present study, female patients were almost twice as many as male, which might explain the difference between genders. It might also suggest that female patients and their parents are more motivated for dental treatment (Lipsky et al. 2021).

There are several studies (Badrov et al. 2017; Dzemidzic et al. 2020; Fekonja. 2015; Pop Acev and Gjorgova 2014; Sola et al. 2018) in agreement with the present study describing the most commonly missing teeth being mandibular second premolars followed by maxillary lateral incisors and maxillary second premolars in that particular order. More studies (Aasheim and Ogaard. 1993; Baron et al. 2018; Pallikaraki et al. 2020; Rølling and Poulsen. 2009; Tallón-Walton et al. 2010) report mandibular second premolars as the most commonly missing tooth group but followed by a different sequence than this study. Four other studies (Drenski Balija et al. 2022; Gábris et al. 2006; Reshitaj et al. 2019; Vinjolli et al. 2023) name upper lateral incisors as the most commonly missing teeth.

The prevalence of one tooth agenesis was the most common condition with nine studies (Aasheim and Ogaard. 1993; Badrov et al. 2017; Baron et al. 2018; Fekonja. 2015; Gracco et al. 2017; Laganà et al. 2017; Magnússon. 1977; Pop Acev and Gjorgova. 2014; Rølling and Poulsen. 2009; Trakinienė et al. 2013) reporting it being present in 40.6–55.1% of the patients, as it was in the present study. Data from a meta-analysis done by Polder and co-authors (Polder et al. 2004) show similar results that most commonly only one tooth is missing (48%). Two studies (conducted in Slovenia and Kosovo (Fekonja. 2015; Reshitaj et al. 2019) report agenesis of two teeth was more common.

Tooth agenesis and the presence of other dental anomalies at the same time were not widely studied among European countries. Fekonja (Fekonja. 2015) reports that 14.3% of Slovenian patients with tooth agenesis had additional dental anomalies (microdontia, impaction of permanent canines, and maxillary canine or first premolar transposition). This is similar to the present study (15.6%). Tallón-Walton and co-authors also describe additional anomalies in Spanish hypodontia patients similar to the present study—supernumerary teeth (0.39%), microdontia (5.5%), peg-shaped teeth (3.47%) and impacted teeth (1.51%). They also report taurodontism (7.1%) and ectopic tooth eruption (3.7%) (Tallón-Walton et al. 2010).

The main limitation of this study should be mentioned that this study was conducted in one dental clinic, not all across the country. Although it is possible that a multicentre study could provide different results, it should be mentioned that Riga Stradins University Institute of Stomatology is the largest dental clinic in Latvia and is attended by patients all over the country.

## Conclusions

This study found that the prevalence of non-syndromic tooth agenesis in Latvian adolescent dental patients was 9.3% with no statistically significant differences between the genders. Patients with tooth agenesis have a statistically significant possibility of the presence of other dental anomalies (*p* < *0.001*).
